# The Cough Response to Inhaled Mannitol in Healthy Subjects

**DOI:** 10.1007/s00408-024-00755-6

**Published:** 2024-11-28

**Authors:** Hanna M. Nurmi, Anne M. Lätti, Heikki O. Koskela

**Affiliations:** 1 Division of Respiratory Medicine, Faculty of Health Sciences, School of Medicine, Institute of Clinical Medicine, University of Eastern Finland, POB 1627, 70211 Kuopio, Finland; 2 Division of Respiratory Medicine, Center of Medicine and Clinical Research, Kuopio University Hospital, POB 100, 70029 Kuopio, Finland

**Keywords:** Cough, Chronic cough, Cough provocation test, Mannitol, Bronchial hyperresponsiveness

## Abstract

**Purpose:**

Inhaled mannitol induces bronchoconstriction and cough. This study aimed to describe the cough response to mannitol among healthy adult subjects.

**Methods:**

125 healthy subjects (aged 18–82 years, 52% females, 50% skin prick test positive) underwent a mannitol test. The coughs were recorded both simultaneously and afterwards from video recordings by two researchers. Three indices were evaluated: The cumulative number of coughs per cumulative dose of mannitol (CDR), cumulative provocative dose of mannitol to cause at least 5 coughs, and the maximal number of coughs provoked by any single mannitol dose. The test was repeated in 26 subjects after 3–7 days.

**Results:**

CDR showed the best repeatability with an intraclass correlation coefficient of 0.829. Gender was the only characteristics that associated with the cough response: The median CDR was 2.53 (interquartile range 0.45–7.01) coughs/100 mg among females and 0.787 (0.0–3.29) coughs/100 mg among males (*p* = 0.002). The interquartile range upper limits were defined as the cut-off limits for a normal response. The threshold for a statistically significant change in CDR was 6.26 coughs/100 mg. There was a close correlation between simultaneous- and video-assessed CDR (intraclass correlation coefficient 0.985).

**Conclusion:**

Females cough more than males in response to mannitol. CDR is the most suitable index to describe the cough responsiveness. The repeatability of the response is good. Video recording of the coughs is not mandatory. The cut-off limits for a normal cough response to mannitol were provided.

**Supplementary Information:**

The online version contains supplementary material available at 10.1007/s00408-024-00755-6.

## Introduction

In many cases of chronic cough, the underlying mechanism is sensitization of the neural pathways that evoke cough, i.e., cough reflex hypersensitivity (CRH) [1, 2]. Cough provocation tests (CPT) can be used to objectively assess the sensitivity of the cough reflex [3]. The wide variability of the response, multiple different tussive agents, lack of standardisation, lack of reference values and issues with feasibility have hindered the clinical use of CPTs [4].

Mannitol inhalation test is a standardized, commercially available, and regulatory approved test to assess airway smooth muscle hyperresponsiveness [5]. Recently, it has also been evaluated to assess CRH. Cough responsiveness to mannitol can separate patients with classic asthma from healthy subjects. In asthma, cough sensitivity to mannitol decreases in parallel with subjectively assessed improvement in chronic cough during therapy with inhaled corticosteroids [6]. Cough responsiveness to mannitol can also separate patients with chronic cough from healthy subjects and patients with other respiratory symptoms [7–9]. As the mannitol test can identify both airway smooth muscle hyperresponsiveness and CRH, it offers the possibility to evaluate two pathophysiological airway phenomena at the same time.

The primary purpose of the present study was to investigate the association of the cough response to mannitol with key subject characteristics among healthy subjects. The secondary purposes were to create cut-off limits for a normal cough response to mannitol, to assess the repeatability of the response, to define the most suitable index to describe the cough sensitivity to mannitol, and to define whether video recording is obligatory to reliably count the coughs.

## Materials and Methods

### Population

The sample size was estimated to reveal the possible associations of the cough responsiveness to mannitol with age and gender, utilizing a linear regression model. The required sample size was minimum 120 subjects.

**Fig. 1:**
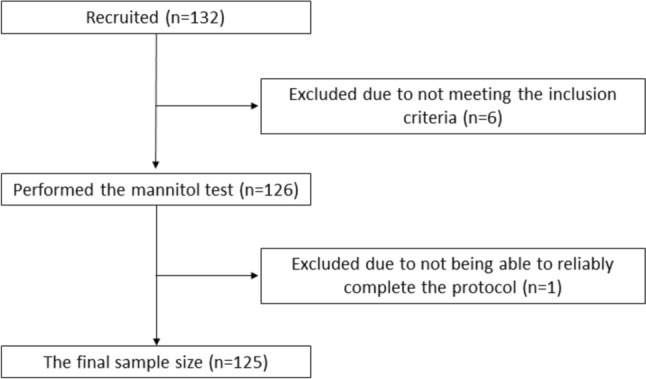
Flow chart

132 subjects were recruited via media and word of mouth. The inclusion criteria encompassed subjects ≥ 18 years old who could understand the purpose of the study. We targeted two even age groups of subjects aged 18–50 and ≥ 51 years, and an even female/male ratio. The exclusion criteria are listed in Table 1. Of the 132 recruited subjects, six were later excluded based on their answers to the health questionnaires and one due to failure of reliably completing the mannitol test (Fig. 1).

**Table 1:** The exclusion criteria

1	Current daily smoking
2	Previous smoking of more than 10 pack years or stopping daily smoking less than a year ago
3	Pregnancy or breastfeeding
4	A doctor´s diagnosis of any respiratory diseases
5	A doctor´s diagnosis of gastro-oesophageal reflux disease
6	Current use of angiotensin-converting enzyme inhibitors
7	Heartburn or regurgitation once a week or more often during the last 3 months
8	Upper respiratory tract infection within 4 weeks
9	Any current (within 4 weeks) cough
10	Chronic (over 2 months´duration) cough during the last 12 months
11	Repeated attacks of shortness of breath or cough at night during the last 12 months
12	Repeated wheezing during the last 12 months
13	Symptoms of chronic (over 3 months´ duration) rhinitis during the last 12 months. This was defined as a positive response to both a or b and c or d of the questions. (a) nasal discharge (anterior or posterior nasal drip) (b) nasal blockage (c) facial pain or pressure (d) reduction/loss of smell
14	Abnormal anatomy or any previous operations to the respiratory organs, which may affect the deposition of the mannitol powder
15	Unable to provide a written informed consent to participate to the study
16	Unable to speak fluent Finnish

### Study Design

All participants filled in a form with questions about their previous respiratory health and symptoms, medication, and smoking history. Symptoms of chronic rhinosinusitis and gastro-oesophageal reflux disease were assessed using standardized questionnaires [10, 11]. Due to technical reasons, not all but 117 participants underwent skin prick tests to examine sensitization to common allergens. Skin test result was considered positive if any wheal diameter was 3 mm greater than the negative control. The result was considered negative if all wheal diameters were less than 3 mm with a positive histamine control [12].

All subjects underwent a mannitol test according to the manufacturer´s recommendations (Aridol™, Pharmaxis Ltd, Sydney Australia). It was video recorded. Spirometry was performed before and during the test according to international recommendations [13] and utilizing local reference values [14]. After finishing the mannitol test, the participants were asked to evaluate how comfortable they experienced the test using a 100 mm visual analogue scale (0 indicating “very comfortable” and 100 mm “extremely uncomfortable”). To investigate the repeatability of the response, twenty-six subjects with a measurable cough response to mannitol (at least 5 coughs in response to any mannitol dose) underwent the mannitol test twice, 3–7 days apart.

### The Mannitol Test

The test started by performing spirometry three times. After that, an empty capsule was inhaled with a 5-seconds breath-holding. A nose clip was used during the inhalation. The test continued with increasing doses of mannitol (5, 10, 20, 40, 2 × 40, 4 × 40, 4 × 40, 4 × 40 mg). One addition to the manufacturer´s instructions was made: In order to standardize inspiratory flow rate, the duration of the inhalation was determined as 3 s [15]. During the test, subjects were instructed not to talk or suppress coughing. 60 s after each dose, spirometry was performed twice. The test was stopped if a ≥ 15% reduction in forced expiratory volume in one second (FEV_1_) was observed, if the subject requested it, or if the maximal cumulative dose of 635 mg was achieved. In case of a ≥ 15% reduction in FEV_1_, 200 mcg salbutamol was administered via spacer (Ventoline Evohaler, GlaxoSmithKline Ltd, London, United Kingdom; OptiChamber Diamond, Philips Medical Systems, Nederland).

### Counting of the Coughs

Cough was defined as a forced expiratory manoeuvre against a closed glottis either with or without preceding inhalation. A trained research nurse counted the coughs manually during the test. Two experienced researchers (H.N. and A.L.) re-counted the coughs afterwards from the video recordings. A timer was started at the beginning of the inhalation of a capsule. All coughs occurring during the following 60 s were recorded after every dose. In higher doses, where multiple capsules were utilized, the coughs occurring between the capsules were counted as well as those occurring within 60 s after the beginning of the inhalation of the last capsule.

### Expression of the Cough and the Bronchoconstrictive Responses to Mannitol

The cough response to mannitol was expressed in three ways. First, as coughs-to-dose ratio (CDR), the cumulative number of coughs per final cumulative dose of mannitol, expressed as coughs per 100 mg mannitol. Second, as the cumulative provocative dose of mannitol to cause at least 5 coughs (PD_5_), calculated by linear interpolation of two consecutive mannitol doses. If PD_5_ was not reached, an arbitrary value of 1270 mg was used for statistical analyses. Third, as the maximal cough response (MAX), calculated as the maximal number of coughs provoked by any single mannitol dose. Each index was composed using the mean value of the two investigators’ assessment of the video recordings. The bronchoconstrictive response to mannitol was expressed as the response-to-dose ratio (RDR), calculated as the final percentage fall in FEV_1_ divided by the final cumulative dose of mannitol [5].

### Statistical Analysis

**Fig. 2:**
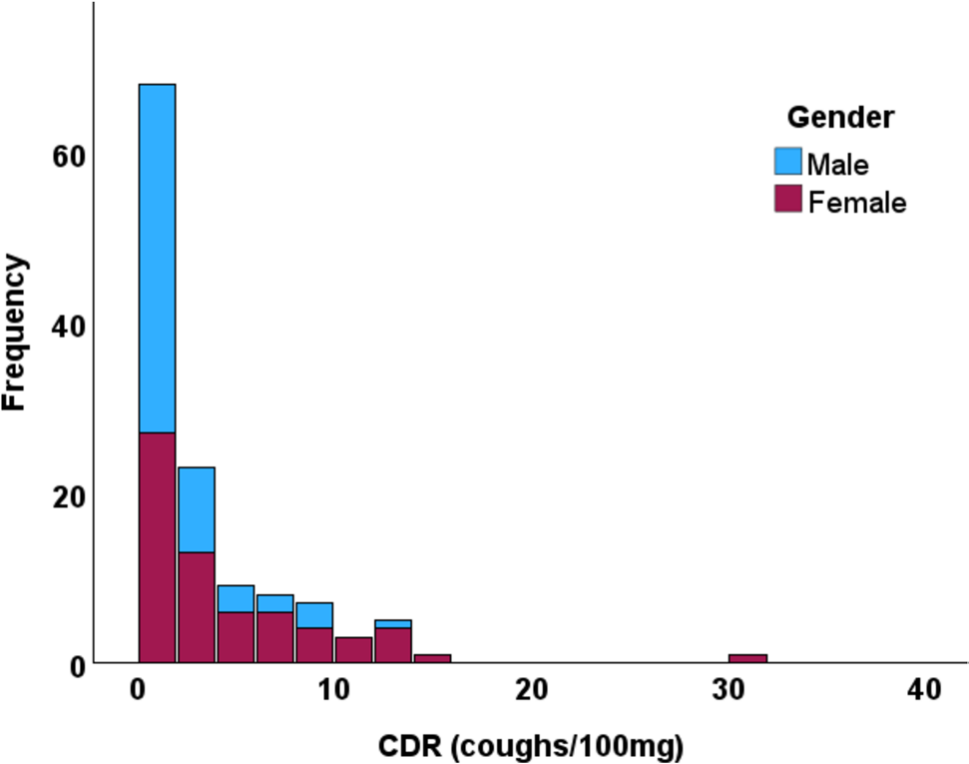
The distribution of coughs-to-dose ratios (CDR) among 125 healthy subjects

The distributions of the cough response indices were verified by the Kolmogorov-Smirnov test. They differed significantly from the normal distribution (Fig. 2). Thus, continuous variables are expressed as medians and interquartile ranges unless stated otherwise. Categorical variables are expressed as numbers and percentages. Chi-square test, Mann-Whitney test, Spearman correlation coefficient (Rs), and multivariate median regression analyses were used when appropriate. The cut-off limits for a normal cough response to mannitol were expressed as the interquartile range upper limits.

The repeatability of the cough indices was evaluated by intraclass correlation coefficient and Bland-Altman plots [16]. The threshold for a statistically significant change in the cough response to mannitol was calculated as 2 x standard deviation of the difference between the two mannitol tests [16]. To investigate the agreement between simultaneous and video cough counting methods, intraclass correlation coefficient was utilized. Data was analysed using SPSS software, version 29.0 (IBM statistics, United States).

## Results

The final sample of 125 subjects had an age range of 18–82 years, with 47.2% of the subjects being > 50 years old, and an almost equal male-female-ratio (Table 2). Four subjects´ test was stopped due to a ≥ 15% fall in FEV_1_ before the maximal mannitol dose and two further subjects had a ≥ 15% fall in FEV_1_ after the maximal dose. All these subjects were included in the cough analyses. The cough responsiveness to mannitol did not correlate with the bronchoconstrictive response (CDR/RDR: Rs 0.02, *p* = 0.80, PD_5_/RDR: Rs 0.04, *p* = 0.70, and MAX/RDR: Rs −0.18, *p* = 0.38).

**Table 2:** Basic characteristics of the subjects

	All (*n* = 125)	Female (*n* = 65 /52%)	(Male (*n* = 60/48%)	*p*-value
Age (years)	49.0 (29–60.5)	49.0 (24.0–60.0)	48.5 (33.3–62.0)	0.23
Ex-smokers	20 (16.0%)	6 (9.2%)	14 (23.3%)	0.032
Pack-years*	0.0 (0.0–0.0)	0.0 (0.0–0.0)	0.0 (0.0–0.0)	0.15
Height (cm)	171 (165–178)	166 (160–170)	178 (173–183)	< 0.001
BMI (kg/m^2^)	24.4 (22.7–27.3)	24.0 (22.2–26.4)	25.4 (23.3–27.7)	0.12
Positive skin prick test**	58/116 (50.0%)	27/58 (46.6%)	31/58 (53.5%)	0.46
Baseline FEV_1_ (% of predicted)	95.0 (88.0–104)	95.0 (86.0–104)	95.5 (89.0–102.8)	0.73

Data is expressed as medians (interquartile ranges) or numbers (percentages)

*BMI* body mass index, *FEV*_1_ forced expiratory volume in one second

*Data missing from six subjects

**Data missing from nine subjects

**Fig. 3:**
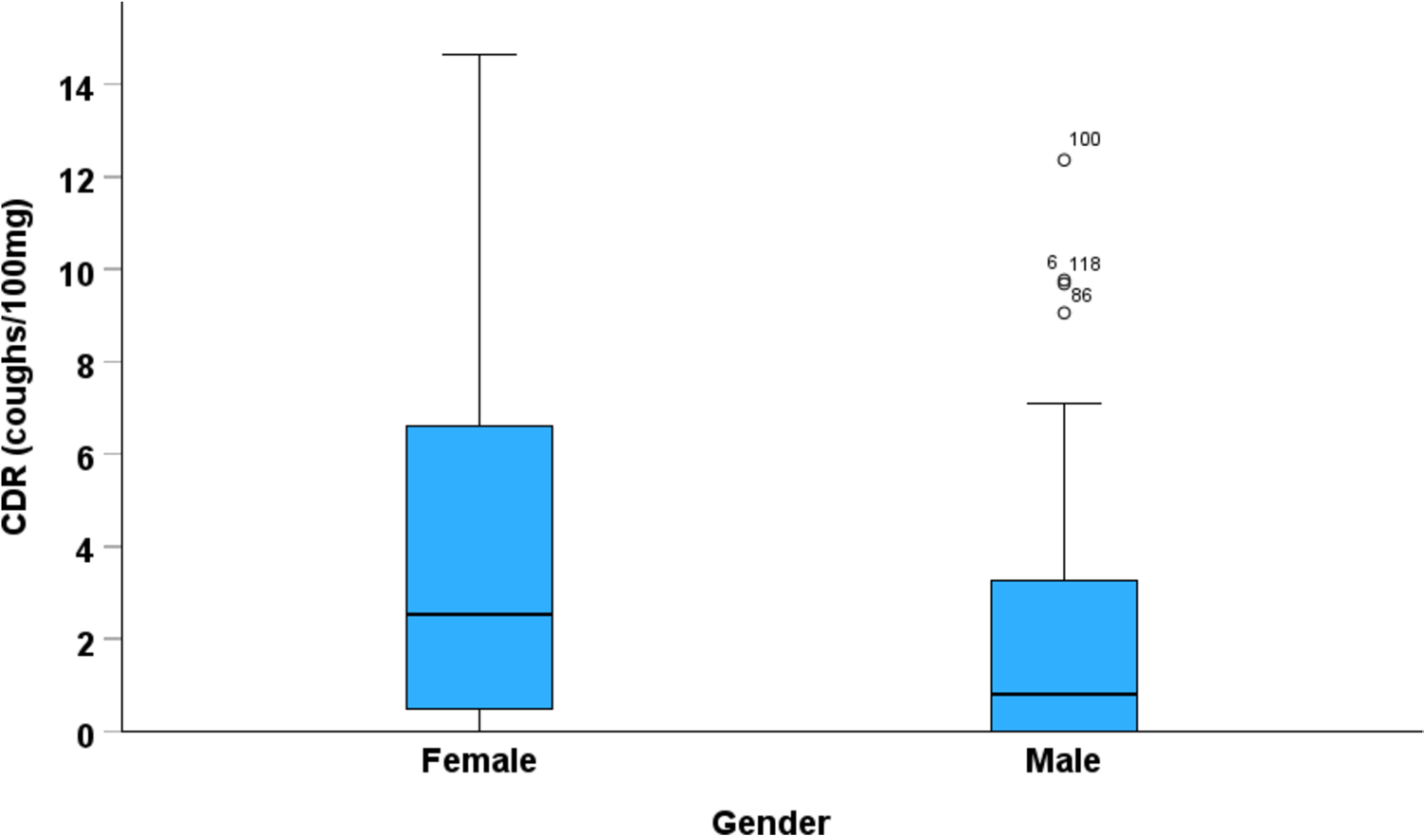
The coughs-to-dose ratios (CDR) among female and male subjects. The upper and lower limits of the blue boxes indicate the interquartile ranges and the horizontal lines in the boxes the medians. The upper and lower limits of the vertical lines indicate the maximum and minimum values that are not outliers. *p* = 0.002 between the genders

**Fig. 4:**
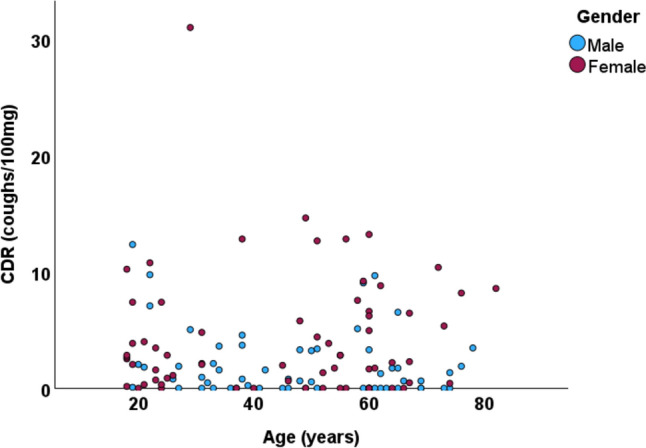
The relation between coughs-to dose ratios (CDR) and age. Spearman’s *r* = − 0.082, *p* = 0.362

In bivariate analyses, cough responsiveness to mannitol was associated with low height, low FEV_1_% predicted, and female gender (Table 3; Figs. 2 and 3). Age was not associated with the responsiveness (Table 3; Fig. 4), not even when the genders were analysed separately (data not shown). In the multivariate median regression models, gender was the only parameter affecting the cough response: Female gender increased the median CDR by 1.74 coughs/100 mg (95% confidence interval 0.440–3.04, *p* = 0.009), decreased the median PD_5_ by 883 mg (739–1028, *p* < 0.001), and increased the median MAX by 4,5 coughs (1.09–7.91, *p* = 0.010) Therefore, the cut-off limits for a normal cough response to mannitol were expressed separately for men and women (Table 4). Women considered the test more uncomfortable than men (visual analogue scale 38.0 (13.5–54.0) mm vs. 22.5 (9.0–41.8) mm, *p* = 0.030, respectively).

**Table 3:** The bivariate associations of baseline characteristics with the cough response indices

	CDR (coughs/100 mg)	PD_5_ (mg)	MAX (coughs)
Age	Rs = − 0.082, *p* = 0.36	Rs = − 0.005, *p* = 0.96	Rs = − 0.074, *p* = 0.41
Height	Rs = − 0.252, *p* = 0.005	Rs = 0.211, *p* = 0.018	Rs = − 0.251, *p* = 0.005
BMI	Rs = − 0.035, *p* = 0.70	Rs = 0.040, *p* = 0.66	Rs = − 0.046, *p* = 0.61
FEV_1_% predicted	Rs = − 0.214, *p* = 0.016	Rs = 0.191, *p* = 0.033	Rs = − 0.200, *p* = 0.025
Gender			
Female (*n* = 65)	2.53 (0.45–7.01)	387 (42.6–1270)	8.00 (2.25–16.3)
Male (*n* = 60)	0.787 (0.0–3.29)	1270 (158–1270)	3.75 (0.00–6.88)
p value	0.002	0.012	0.002
Skin prick test*			
Positive (*n* = 58)	2.01 (0.00–3.86)	496 (96.9–1270)	5.25 (0.00–10.0)
Negative (*n* = 58)	1.57 (0.138–6.56)	627 (64.1–1270)	5.00 (0.875–14.6)
p value	0.60	0.89	0.70

The values for genders and skin test groups are expressed as medians (interquartile ranges)

*CDR* coughs-to-dose ratio, *PD*_5_ cumulative provocative dose of mannitol to cause 5 coughs, *MAX* maximal number of coughs provoked by any single mannitol dose, *BMI* body mass index, *FEV*_1_ forced expiratory volume in one second, *Rs* Spearman correlation coefficient

*Data missing from 9 subjects

**Table 4:** The cut-off limits for a normal cough response to mannitol

Index	Gender	Cut-off limit
CDR	Female Male	< 7.01 coughs/100 mg < 3.29 coughs/100 mg
PD_5_	Female Male	> 42.6 mg > 158 mg
MAX	Female Male	< 16 coughs < 7 coughs

*CDR * coughs-to-dose ratio, *PD*_5_ cumulative provocative dose of mannitol to cause 5 coughs, *MAX * maximal number of coughs provoked by any single mannitol dose

**Fig. 5:**
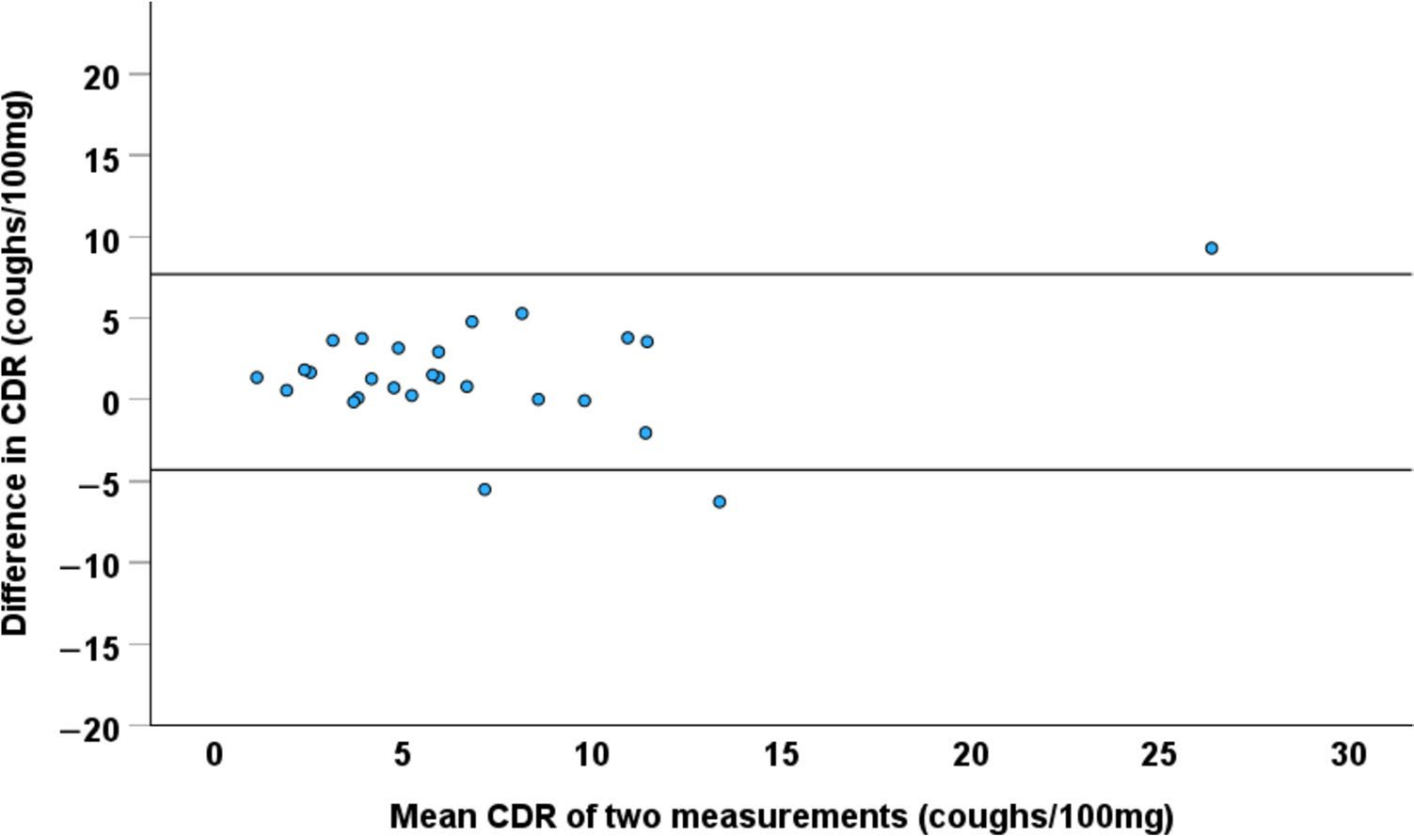
A Bland–Altman plot for coughs-to-dose ratio (CDR) to express its repeatability in two mannitol tests performed 3–7 days apart in 26 healthy subjects. The horizontal lines indicate +/− 2SD of the difference between the two tests

The repeatability of the three cough response indices is shown in Table 5. CDR showed the highest repeatability. It tended to be worse among those with the largest CDR values (Fig. 5). The threshold for a statistically significant change in CDR was 6.26 coughs/100 mg.

**Table 5:** The repeatability of the cough response indices among 26 healthy subjects who underwent the mannitol test twice

Index	First test	Second test	Intraclass correlation coefficient
CDR (coughs /100 mg)	6.49 (4.27–9.88)	4.76 (2.99–9.21)	0.829
PD_5_ (mg)	62.1 (22.2–218)	79.1 (30.4–244)	0.445
MAX (coughs)	13.5 (9.38–18.1)	9.00 (6.00–18.0)	0.687

Data is shown as medians (interquartile ranges)

*CDR * coughs-to-dose ratio, *PD*_5_ cumulative provocative dose of mannitol to cause 5 coughs, *MAX* maximal number of coughs provoked by any single mannitol dose

The simultaneously counted coughs correlated well with the those counted from the videos. The intraclass correlation coefficients between the cough recording methods were 0.985, 0.900, and 0.971 for CDR, PD_5_, and MAX, respectively. A supplementary file including cough response indices of every participant together with age and gender information is provided.

## Discussion

In the present study utilizing a large sample of healthy subjects with a wide age range, only gender affected the cough responsiveness to mannitol in the multivariate models. In our previous studies, female subjects with chronic cough or other chronic respiratory symptoms have also been more responsive to the cough-provoking effect of mannitol than their male counterparts [8, 9]. Women are more responsive than men also to hypertonic saline [17], citric acid [9, 18, 19], tartaric acid [20], and capsaicin [18, 21, 22]. The potential anatomic sites driving CRH include peripheral afferent nerve terminals in the airways and the central nervous system [23]. The apparently stimulus-nonspecific nature of this gender difference suggests that it more probably involves gender-related differences in the processing of cough stimuli in the central nervous system than differences in the function of the peripheral afferent nerve terminals.

Despite the very wide age range of the present sample, no association was found between the cough sensitivity to mannitol and age. Accordingly, in a previous study among 312 patients with chronic cough, no association between capsaicin cough responsiveness and age could be found [24]. However, in a study with 134 healthy subjects, a weak (*r* = − 0.18), but statistically significant association was found between the capsaicin responsiveness and age [21]. In a large study applying capsaicin test in 607 subjects including both healthy subjects and those with chronic cough, men aged 50–89 years were slightly more responsive to capsaicin than those under 50 years of age. However, such an age difference could not be seen among women [22]. Taken together, age does not seem to be a major factor affecting cough reflex sensitivity in adults.

Skin prick test positivity did not associate with the cough sensitivity to mannitol. This is in line with previous studies. In a study with 134 healthy subjects, skin test positivity was not associated with capsaicin cough responsiveness [21]. In a study among 312 subjects with chronic cough and 100 age-matched healthy controls, the percentage of positive skin tests was similar in patients with enhanced capsaicin sensitivity and those with normal sensitivity [24]. It may be concluded that atopy, defined as skin test positivity, does not affect cough reflex sensitivity.

The prevalence of chronic cough may be higher among obese individuals [25]. However, BMI was not associated with cough responsiveness to mannitol. This is in accordance with smaller studies utilizing capsaicin test among healthy subjects, subjects with asthma, or those with airway symptoms elicited by odorous chemicals [26, 27].

This study was first to report the repeatability of the mannitol cough response. Of the three indices, CDR showed the best repeatability with an intraclass correlation coefficient of 0.829, which can be regarded as good [28]. The statistically significant threshold value for a change in CDR was 6.26 coughs/100 mg. However, our healthy subjects tended to show mild cough responses to mannitol and the repeatability seemed to worsen towards higher CDR values. Therefore, this threshold value should be used cautiously, and the repeatability of the cough response to mannitol should be investigated among subjects with stable chronic cough in the future.

Of the different cough response indices, we recommend CDR for clinical use. It showed the best repeatability and the highest agreement between simultaneous and video counting of the coughs. It is easy to calculate. Moreover, in our previous study, its diagnostic performance in identifying subjects with chronic cough was good and slightly better than that of PD_5_ [9].

In this study we have, for the first time, provided the cut-off limits for a normal cough response to mannitol. Since the indices did not distribute normally, the upper limits of the interquartile ranges were chosen instead of the commonly used mean ± 1.96 SD. In our previous study, the CDR cut-off value that had the best diagnostic performance to separate subjects with chronic cough from healthy controls was 5.35 coughs/100 mg [29]. This value is close to the cut-off limits for a normal cough response reported here. Therefore, the now presented cut-off limits for a normal cough response to mannitol may also be used to separate patients with chronic cough from healthy subjects.

Previous studies in various patient groups have shown that the bronchoconstrictive and the cough responsiveness to mannitol do not correlate [6, 9, 30]. This was true also in healthy subjects, corroborating separate mechanisms behind the response types. Despite this, one may think that mannitol-provoked bronchoconstriction might cause cough that is not attributable to CRH. However, bronchoconstriction is generally a weak stimulus for cough [31]. Patients with classic asthma have an especially low cough response to bronchoconstriction [32]. In classic asthma, coughing during mannitol challenge occurs independently of bronchoconstriction [6]. Patients with cough-variant asthma may be an exception. They are especially sensitive to the cough-provoking effect of bronchoconstriction [33]. In these patients, severe coughing during a mannitol test may not necessarily indicate cough sensitivity to mannitol but cough due to mannitol-provoked bronchoconstriction.

The simplicity of the mannitol test makes it feasible in clinical use. It is administrated with a handheld dry powder inhaler, without the need to prepare fresh solutions. A nebulizer or any other special equipment is not needed. The method of administration is standardized. Moreover, it already has regular approvement for assessment of airway hyperresponsiveness and is commercially available. The feasibility of the test is further supported by the present and previously reported [34] evidence that simultaneous cough counting correlates well with video recordings. Therefore, video analysis of the coughs is not mandatory.

The main limitation of the study is the single centre setting. All study subjects were Caucasians. Therefore, the results may not be generalized to apply to other ethnicities. The effect of current smoking was not investigated. The strengths of the present study include explicit exclusion criteria, the double counting of coughs from the video recordings, widespread age range and equal gender and atopy distribution of the subjects, the reproducibility analysis, and the standardized duration of mannitol inhalation.

In conclusion, the present study described the cough response to inhaled mannitol in a large sample of healthy subjects. Gender was the only characteristic that associated with the response. The cough response was repeatable. Video recording of the coughs is not mandatory. The cut-off limits for a normal cough response to mannitol provide an opportunity to measure cough reflex sensitivity and airway smooth muscle hyperresponsiveness simultaneously during mannitol test even in everyday clinical work.

##  Supplementary Information

Below is the link to the electronic supplementary material.

Supplementary file 1 (DOCX 161kb)

## Data Availability

A supplementary file including cough response indices of every participant together with age and gender information is provided.
