# Supplementary material for: The Cough Response to Inhaled Mannitol in Healthy Subjects
Source: Lung. 2024 Nov 28;203(1):5. doi: 10.1007/s00408-024-00755-6 (PMC11604684; doi:10.1007/s00408-024-00755-6)
Supplement: Supplementary file 1 — (DOCX 161kb) [file 408_2024_755_MOESM1_ESM.pdf]

Supplementary data.
Cough response indices of each subject. Gender (f = female, m = male) and age are provided as well.

**Subject**	**Gender**	**Age**	**CDR (coughs/100mg)**	**MAX (coughs)**	**PD5 (mg)**
1	f	74	0,421	2	1270
2	f	21	4	3	1270
3	f	18	2,526	4	1270
4	f	48	5,789	10	2,24
5	f	60	0	0	1270
6	f	26	1,102	3	1270
7	f	20	0	0	1270
8	f	18	2,677	7	475
9	f	51	12,677	28	5
10	f	18	0,157	1	1270
11	f	49	14,646	20,5	4,32
12	f	76	8,189	15	9,94
13	f	19	2,047	4	1270
14	f	25	2,835	8	386,81
15	f	62	8,819	17	42,89
16	f	60	1,654	9,5	553,42
17	f	64	0	0	1270
18	f	21	0,315	2	1270
19	f	31	2,126	10,5	208,93
20	f	60	6,22	16,5	42,35
21	f	23	3,465	10	516,08
22	f	52	1,339	5	635
23	f	56	12,835	19,5	24,02
24	f	52	0	0	1270
25	f	82	8,583	13	107,82
26	f	67	2,283	14,5	362,93
27	f	73	5,354	9	361,22
28	f	51	4,409	9	19,9
29	f	66	0	0	1270
30	f	72	10,394	19,5	16,4
31	f	24	0	0	1270
32	f	40	0	0	1270
33	f	67	6,457	10	22,91
34	f	53	3,858	9	107,82
35	f	18	10,236	19	83,2
36	f	54	1,732	3,5	1270
37	f	31	4,803	23,5	422,4
38	f	19	7,402	10	39,74
39	f	37	0	0	1270
40	f	24	7,402	16,5	21,35
41	f	24	0,315	2	1270
42	f	19	3,858	8	232,44
43	f	18	2,835	10	443,57
44	f	49	0	0	1270

45	f	56	0	0	1270
46	f	31	2,047	6	155
47	f	22	10,787	16	58,17
48	f	23	0,709	2,5	1270
49	f	55	2,835	6	129,27
50	f	45	1,969	5,5	68,18
51	f	61	1,732	6	3,82
52	f	60	13,228	21,5	22,46
53	f	64	2,205	8	279,89
54	f	55	0	0	1270
55	f	29	31,024	39,5	1,82
56	f	60	4,961	18	217,26
57	f	60	6,614	17	171,53
58	f	60	0	0	1270
59	f	67	0,472	2,5	1270
60	f	38	12,835	18,5	39,18
61	f	23	1,575	10	549,2
62	f	59	9,213	17	26,39
63	f	25	0,866	5,5	618,46
64	f	58	7,559	20,5	109,9
65	f	46	0,63	4	1270
66	m	62	0	0	1270
67	m	29	5,039	11,5	220,96
68	m	46	0,787	2	1270
69	m	19	0,079	0,5	1270
70	m	27	1,89	4	1270
71	m	59	9,055	17,5	101,49
72	m	76	1,89	4	1270
73	m	78	3,465	7	30,39
74	m	61	0	0	1270
75	m	32	0,472	3	1270
76	m	26	0,787	5	635
77	m	34	1,575	4	1270
78	m	45	0	0	1270
79	m	36	0	0	1270
80	m	69	0	0	1270
81	m	74	0	0	1270
82	m	59	0	0	1270
83	m	51	3,386	10	30,39
84	m	73	0	0	1270
85	m	64	1,732	5	155
86	m	22	9,764	17	19,9
87	m	19	12,362	31	19,6
88	m	38	4,567	14	166,39
89	m	49	0	0	1270
90	m	69	0	0	1270
91	m	60	0	0	1270
92	m	69	0,63	3	1270
93	m	66	0,63	2	1270
94	m	22	7,087	14	35

95	m	27	0	0	1270
96	m	60	3,307	11	213,95
97	m	60	0	0	1270
98	m	33	0	0	1270
99	m	20	2,047	5	35
100	m	37	0	0	1270
101	m	63	0	0	1270
102	m	31	0,945	6	605,01
103	m	41	0	0	1270
104	m	46	0	0	1270
105	m	50	3,228	6,5	66,05
106	m	61	9,685	17,5	58,17
107	m	62	1,26	4	1270
108	m	38	0,787	5	35
109	m	31	0	0	1270
110	m	34	3,622	10	220,96
111	m	58	5,118	9,5	192,79
112	m	33	2,126	4	1270
113	m	48	3,307	8,5	492,55
114	m	39	0,236	1,5	1270
115	m	65	1,732	5	155
116	m	50	0,551	3,5	1270
117	m	74	1,339	4,5	1270
118	m	38	3,701	12,5	27,47
119	m	21	1,811	6	475
120	m	48	0,63	1	1270
121	m	65	6,535	13	66,05
122	m	55	2,835	5	75
123	m	65	0	0	1270
124	m	51	0	0	1270
125	m	42	1,575	3	1270
